# Healthy Eating Index-2010 and food groups consumed by US adults who meet or exceed fiber intake recommendations NHANES 2001–2010

**DOI:** 10.3402/fnr.v60.29977

**Published:** 2016-04-19

**Authors:** Carla R. McGill, Anne Birkett, Victor L. Fulgonii III

**Affiliations:** 1 Healthy Science Communications LLC, Sarasota, FL, USA; 2 Kellogg Company, Battle Creek, MI, USA; 3 Nutrition Impact LLC, Battle Creek, MI, USA

**Keywords:** dietary fiber, food sources, nutrition surveys, NHANES

## Abstract

**Background:**

The proportion of the US adult population who meet fiber intake recommendations is very low. Information about food groups consumed and diet quality for the adults who consume recommended amounts of fiber are scarce.

**Objective:**

To examine food groups consumed and Healthy Eating Index (HEI-2010) scores for US adults meeting the fiber adequate intake (AI) based on National Health and Nutrition Examination Survey (NHANES) data 2001–2010.

**Design:**

A secondary analysis of NHANES data from 2001 to 2010. Participants included adults aged 19 and older (*n*=24,807) with complete day 1 dietary records. Variables measured were food group sources of fiber and HEI-2010 scores. Sample-weighted data were used to calculate least square means (LSM)±standard error of the mean (SEM) by fiber intake quartile along with HEI-2010 scores. Significance was set at *P*<0.05.

**Results:**

Major fiber food sources for US adults meeting the AI were grain products, vegetables, legumes, and fruits. The top grain products consumed were grain mixtures, ready-to-eat (RTE) cereals, and breads/rolls. The mean HEI-2010 score for adults meeting the AI for fiber was significantly (*P*<0.001) higher compared with all adult participants. The mean HEI-2010 score increased with increasing fiber intake in both groups.

**Conclusions:**

Adults who meet the AI for fiber have a higher quality diet. Fiber may be an important dietary component that predicts diet quality.

Dietary fiber was identified as a nutrient of concern in the 2010 Dietary Guidelines for Americans (DGA 2010) based on the very low dietary intakes across all sectors of the US population and its important contribution to health (1). Adequate intake (AI) values for fiber intake recommendations for adults are 38 g/day for males aged 19–50, 30 g/day for males aged 51 and older, 25 g/day for females aged 19–50, and 21 g/day for females aged 51 and older (2). Recent reports of fiber intakes based on National Health and Nutrition Examination Survey (NHANES) data are below these recommendations. Reicks et al. reported that the mean fiber intake for US adults aged 19 and older was 17.0 g/day based on NHANES 2009–2010 data (3). An analysis of 2001–2010 NHANES data reported the mean fiber intake for adults aged 19 and older as 16.1 g/day (4). A previous analysis of 1999–2010 NHANES data reported fiber intakes for adults aged 20 and older as 15.7–17.0 g/day (5). The main food sources of dietary fiber based on NHANES 2001–2010 data were vegetables (22.6%), other foods (14.3%), grain mixtures (12.0%), and fruits (11.1%) (4). The percentage of US adults who have an AI of fiber is low. The Institute of Medicine (IOM) calculated that less than 10% of any age group had fiber intakes greater than the AI (2). Others have reported the percentage of the population meeting the AI for fiber to be as low as 5% (6) and 3% (7). Data are scarce regarding food sources of fiber for US adults who meet or exceed the AI values for fiber intake.

The Healthy Eating Index (HEI) is a diet quality index that measures conformance to federal dietary guidance (8). It is used to monitor the quality of American diets and is a metric that can be applied to any defined set of foods (9). The HEI-2010 reflects the DGA 2010 and is made up of 12 components that are summed to provide a total score that has a maximum of 100 points (9). The mean HEI-2010 score for the US population aged 2 and older was 53.5 based on 2007–2008 NHANES data (9). The low mean HEI-2010 score for the US population is a reflection of the overall food supply and is of concern since higher HEI-2010 scores reflect a higher quality diet and have been associated with lower mortality in men and women (10–12). To date, there are no reports of HEI-2010 scores for US adults who meet or exceed the AI values for fiber intake.

The purpose of this study was to examine the food groups consumed and HEI-2010 score for US adults meeting or exceeding the AI for fiber over the 10-year period from 2001 to 2010 based on NHANES data.

## Methods

### Study design and population

The NHANES is a cross-sectional survey that collects data about the nutrition and health status of the US population using a complex, multistage, probability sampling design (13). The NHANES is conducted in a noninstitutionalized, civilian US population by the National Center for Health Statistics (NCHS). Participants of NHANES completed a comprehensive questionnaire assessing dietary behaviors, health history, socioeconomic status, and demographic information at NHANES Mobile Examination Centers and in participants’ homes. The NCHS Research Ethics Review Board reviewed and approved all study protocols for NHANES 2001–2010. The analysis described here was a secondary analysis that lacked personal identifiers; therefore, this study did not require institutional review.

Data are released in two-year increments, and for this analysis, data cycles from 2001 through 2010 were combined. Data from adults aged 19 and older (*n*=24,807) were included. Analyses included only individuals with complete and reliable dietary records as determined by the NCHS staff and excluded females who were pregnant or lactating (13).

Demographic information, including age, gender, and race-ethnicity, used for covariates in the statistical analyses outlined below, was determined via interview (13).

### Dietary assessment

Trained interviewers conducted in-person 24-h dietary recalls using the US Department of Agriculture's (USDA's) Automated Multiple-Pass Method (14, 15). Dietary data included detailed descriptions of all food and quantities eaten. Detailed descriptions of the dietary interview methods are provided in the NHANES Dietary Interviewer's Training Manual (13). Dietary intake data from day 1 were used for analysis in this study. Total dietary fiber is a variable reported in NHANES and is based on values reported in the USDA's Food and Nutrient Database for Dietary Studies (FNDDS). The AI values used in the analyses were the current recommendations from the IOM based on age and gender: 38 g for males aged 19–51, 30 g for males aged 51+, 25 g for females aged 19–51, and 21 g for females aged 51+ (2).

### Data analysis and HEI-2010 score calculation

Sample-weighted data were used in all statistical analyses, and all analyses were performed using SAS 9.2 with SUDAAN Release 11 (Research Triangle Institute, Research Triangle Park, NC). Subjects meeting or exceeding the AI for fiber were identified and their diets specifically analyzed to identify sources of fiber in their diet. In a separate analysis, subjects were also placed into quartiles of fiber intake. Least square means (LSM)±standard error of the mean (SEM) by fiber intake quartile were calculated using the fiber quartile calculated from the specific population being analyzed. The covariates used in the regressions were age, gender, and ethnicity. A *P* value of <0.05 was considered significant.

The 12 components of the HEI-2010 score were calculated using NHANES day 1 dietary data and day 1 dietary weights. Assigning HEI-2010 scores to a set of foods requires translating them into amounts of food groups that are consistent with the USDA Food Patterns (16). The scores of the 12 components are summed to yield a total score with a maximum value of 100.

## Results

Table 1 presents food group sources of fiber for adults aged 19+ who met the gender- and age-appropriate AI for fiber intake. The largest food group contributor to fiber intake was grain products (40.6%), followed by vegetables (17.8%), dry beans, peas, legumes, nuts and seeds (17.1%), and fruits (15.6%). Combining these foods groups contributed 91.1% of the fiber in US adult diets meeting or exceeding the AI for fiber.

**Table 1:** Food sources of fiber for US adults aged 19+ meeting or exceeding the AI for fiber intake NHANES 2001–2010 (*n*=2,565)

Ranking	Food/food group	% total	Cumulative %
1	Grain products	40.6	40.6
2	Vegetables	17.8	58.4
3	Dry beans, peas, legumes, nuts, and seeds	17.1	75.5
4	Fruits	15.6	91.1
5	Meat, poultry, fish, and mixtures	4.8	95.9
6	All other foods	4.1	100

**Fig. 1:**
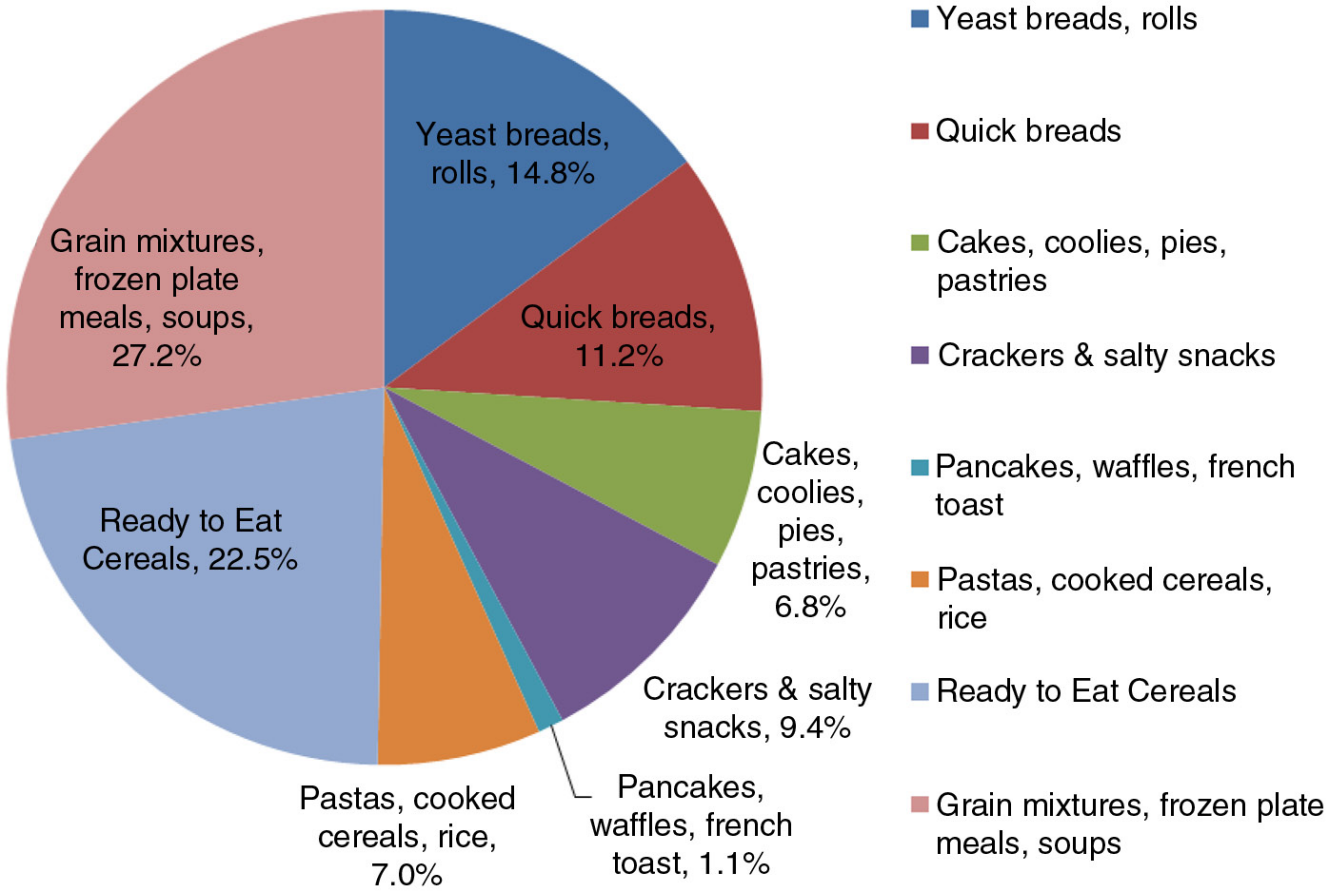
Grain products consumed by US adults aged 19+ meeting the AI for fiber NHANES 2001–2010 (*n*=2,565).

Grain products consumed by adults aged 19+ meeting or exceeding the AI for fiber are illustrated in the Fig. 1. Grain mixtures, frozen plate meals, and soups contributed the largest percentage of fiber (27.2%), followed by ready-to-eat (RTE) cereals (22.5%), yeast breads, rolls (14.8%), quick breads (11.2%), and crackers and salty snacks (9.3%). These products contributed 85% of the fiber from grain products to the diets of US adults meeting or exceeding the AI for fiber. Pastas, cooked cereals, and rice contributed an additional 7% of fiber followed by cakes, cookies, pies, and pastries, which contributed 6.7% of fiber intake.

The total HEI-2010 score and changes in the score components by increasing fiber intake quartile for adults aged 19+ who met the AI for fiber are presented in Table 2. The total HEI-2010 score was 61.05 and the total score increased 3.10 points for each quartile increase in fiber intake. Table 3 presents the total HEI-2010 score for all adults aged 19+ and changes in the score components by increasing fiber intake quartile. The total HEI-2010 score was 47.79 and the total score increased 5.79 points for each quartile increase in fiber intake. The mean fiber intake for all adults aged 19+ from NHANES 2001–2010 was 16.1 g/day (data not shown; reported by McGill et al. (4)). The total HEI-2010 score for adults meeting or exceeding the AI for fiber was significantly (*P*<0.001) higher than the score for all adults aged 19+ (Tables 2 and 3).

**Table 2:** HEI-2010 score for adults aged 19+ meeting or exceeding the AI for fiber and changes in HEI-2010 score components by increasing fiber quartile NHANES 2001–2010 (*n*=2,565)

HEI 2010 component	Contribution to total score Mean±SE	Change in score by increasing fiber quartile
Total vegetables	3.86±0.04	0.09
Greens and beans	2.53±0.06	0.29
Total fruit	3.23±0.06	0.11
Whole fruit	3.39±0.06	0.12
Whole grains	3.97±0.12	0.41
Dairy	5.21±0.10	−0.02
Total protein foods	4.24±0.04	0.11
Seafood and plant protein	3.15±0.06	0.40
Fatty acid ratio	5.98±0.09	0.58
Sodium	4.31±0.08	−0.03
Refined grains	6.46±0.09	0.16
SoFAAS calories	14.72±0.15	0.87
Total score	61.05*±0.38	3.10

SE, standard error; SoFAAS, calories from solid fats, alcohol and added sugars.

* *P*<0.001 compared with total score for all adults in Table 3 by *t*-test.

**Table 3:** HEI-2010 score for all adults aged 19+ and changes in HEI-2010 score components by increasing fiber quartile NHANES 2001–2010 (*n*=24,807)

HEI 2010 component	Contribution to total score Mean±SE	Change in score by increasing fiber quartile
Total vegetables	3.04±0.01	0.49
Greens and beans	1.18±0.02	0.54
Total fruit	2.14±0.03	0.55
Whole fruit	2.01±0.02	0.70
Whole grains	2.19±0.03	0.77
Dairy	5.02±0.04	0.19
Total protein foods	4.16±0.01	0.04
Seafood and plant protein	1.93±0.02	0.53
Fatty acid ratio	4.89±0.04	0.41
Sodium	4.26±0.03	−0.13
Refined grains	6.03±0.05	−0.01
SoFAAS calories	10.96±0.09	1.70
Total score	47.79*±0.19	5.79

SE, standard error; SoFAAS, calories from solid fats, alcohol and added sugars.

* *P*<0.001 compared with total HEI score for adults meeting fiber AI in Table 2 by *t*-test.

## Discussion

Among US adults who participated in NHANES from 2001 to 2010 (*n*=24,807), the percentage who met the AI for fiber intake was 10.3% (*n*=2,565). This result is similar to the IOM Dietary Reference Intake report which stated that based on the AI set for various age and gender groups, 10 percent or less of a particular group had fiber intake greater than the AI (2). Others have reported lower percentages, 3–5%, of the US population meeting the AI for fiber intake (6, 7). The differences in reported percentages of the population meeting the fiber AI may be due in part to different sample sizes and different NHANES data cycles used for the calculation.

Food group sources of fiber for adults meeting or exceeding the AI were slightly different from sources of fiber reported by others for the US adult population. Grain products were the food group contributing the most fiber for those meeting the AI, followed by vegetables, dry beans/peas/legumes/nuts/seeds, and fruit. Previous reports of top sources of fiber for US adults were vegetables and fruit (3), vegetables and other foods (4), and yeast bread/rolls and fruit (17). The top three grain products consumed by adults meeting or exceeding the AI were grain mixtures/frozen plate meals/soups, RTE cereals, and yeast breads/rolls. There are currently no previous reports of the major sources of grain products consumed by adults who meet or exceed the fiber AI in the literature.

The HEI-2010 score is a valid and reliable measure of diet quality (8). The HEI-2010 has 12 components and assesses dietary intakes on the basis of density rather than quantity. Nine of the components are food groups or dietary components to encourage and higher intakes result in higher scores. For three of the components, refined grains, sodium, and empty calories (calories from solid fats, added sugars, and alcohol), lower intake levels result in higher scores. The maximum HEI-2010 score is 100. Reported HEI-2010 scores for the US population are low. Scores for the US population aged 2 and older have been reported to be 49.9 (8), or 51.9 and 53.5 (9) depending on which NHANES data cycle was used to calculate the score. When the HEI-2010 score is applied to US food supply data, the score ranges from 48 points in 1970 to 55 points in 2010 (10). Higher HEI-2010 scores are indicative of a higher quality diet and have been associated with reduced risk of all-cause, cardiovascular and cancer mortality in men and women (11, 12, 18, 19).

To the authors’ knowledge, this study is the first to report the HEI-2010 score for US adults who meet or exceed current fiber intake recommendations based on the AI. The mean HEI-2010 score for US adults meeting or exceeding the AI for fiber intake was significantly higher (27.7% higher) than the score for all adults aged 19 and older participating in NHANES 2001–2010. Scores for all 12 components of the total HEI-2010 score were higher among the adults meeting the AI for fiber compared with all adult participants (Tables 2 and 3). This indicates that adults who meet or exceed the AI for fiber intake have a higher quality diet overall. Adults who meet the AI for fiber intake may select healthier options within the food groups that are components of the HEI-2010 score.

The HEI-2010 score increased for each increase in fiber intake quartile for all US adults and for those meeting the AI for fiber intake, indicating that fiber is a diet component that may predict diet quality or at least is associated with a higher quality diet. The increase in total HEI-2010 score for all adults by increasing fiber quartile was nearly twice that of the increase for those adults meeting the AI for fiber intake (5.79 vs. 3.10). This indicates that fiber intake may be an important marker of diet quality at intake levels below current recommendations. These data also suggest that even small changes in fiber intake are associated with large changes in HEI-2010 score for all adults; the smaller increase seen in those that meet or exceed the AI for fiber is probably due to the fact this group had higher HEI-2001 scores even in the lower quartiles of intake.

Limitations to this study include the cross-sectional design of NHANES, which does not allow causal inferences. In addition, a single 24-h dietary recall may not reflect the usual dietary pattern of participants and may under- or over-report food intake. However, NHANES is a large observational study of a nationally representative sample of the US population that allows the assessment of numerous outcomes.

## Implications for research and practice

Based on NHANES 2001–2010, fiber intake by US adults is below recommendations. The small percentage of adults who met or exceeded fiber intake recommendations had a significantly higher HEI-2010 score and a higher quality diet overall. Foods that contribute fiber to the diet of adults who met the AI were grain products (grain mixtures, RTE cereals, and breads/rolls), vegetables, legumes, and fruits. The mean HEI-2010 score, an indicator of diet quality, significantly increased with increasing fiber intake. Fiber may be an important diet component that indicates diet quality. Increasing fiber intake for the US adult population would increase diet quality and may reduce risk of several chronic diseases.
